# On-line glucose monitoring by near infrared spectroscopy during the scale up steps of mammalian cell cultivation process development

**DOI:** 10.1007/s00449-019-02091-z

**Published:** 2019-02-26

**Authors:** Bence Kozma, András Salgó, Szilveszter Gergely

**Affiliations:** 1 Department of Applied Biotechnology and Food Science, Budapest University of Technology and Economics, Műegyetem rkp. 3, Budapest, 1111 Hungary; 2 Biotechnology Development Department, Gedeon Richter Plc, Gyömrői út 19-21, Budapest, 1103 Hungary

**Keywords:** NIR, Glucose, PCA, PLS, Monitoring, Mammalian cells

## Abstract

NIR spectroscopy is a non-destructive tool for in-situ, on-line bioprocess monitoring. One of its most frequent applications is the determination of metabolites during cultivation, especially glucose. Previous studies have usually investigated the applicability of Near Infrared (NIR) spectroscopy at one bioreactor scale but the effect of scale up was not explored. In this study, the complete scale up from shake flask (1 L) through 20 L, 100 L and 1000 L up to 5000 L bioreactor volume level was monitored with on-line NIR spectroscopy. The differences between runs and scales were examined using principal component analysis. The bioreactor runs were relatively similar regardless of scales but the shake flasks differed strongly from bioreactor runs. The glucose concentration throughout five 5000 L scale bioreactor runs were predicted by partial least squares regression models that were based on pre-processed spectra of bioreactor runs and combinations of them. The model that produced the lowest error of prediction (4.18 mM on a 29 mM concentration range) for all five runs in the prediction set was based on the combination of 20 L and 100 L data. This result demonstrated the capabilities and the limitations of an NIR system for glucose monitoring in mammalian cell cultivations.

## Introduction

Chinese hamster ovary (CHO) cells are the mammalian workhorse of the biopharma industry. They are extensively used for recombinant protein production, especially monoclonal antibodies (mAb) [1, 2]. Classical recombinant protein production is usually carried out with bacterial (e.g. *E. coli*) or yeast cells (e.g. *P. Pastoris*) but complex protein molecules, which have several post-translational modifications to function properly as a medicine, require mammalian host cells [3]. However, these cells are more sensitive to cultivation parameters than the above-mentioned bacterial or yeast cells because they do not possess a cell wall that reinforces the cells. Therefore, strict monitoring and control of the cultivation parameters is mandatory to ensure process reproducibility and the desired product quality [4]. In 2004, the US Food and Drug Administration (FDA) released the guidance of process analytical technology [5] that encouraged the manufacturers to use on-line measurement techniques to monitor and control the cultivation parameters. The concept of process analytical technology was shortly followed by quality by design that emphasized the designing of processes with systematic experiments (i.e. Design of Experiments) to discover the behaviour of the processes according to a set of different cultivation parameters. The parameters that are proven to affect the critical quality attributes (CQAs) of the protein product are the critical process parameters (CPPs) that have to be monitored and controlled during the process to ensure the desired product quality.

Glucose is the main carbon and energy source of the cells that is consumed during the cell cultivation process and then turned into biomass and the product [4, 6]. However, the availability of glucose to the cells influences several CQAs because it could alter cell metabolism [7]. Thus, glucose concentration is usually a critical process parameter that is generally controlled between 5 and 40 mM during a cultivation process. On the one hand, glucose concentration lower than 5 mM can lead to the starvation of the cells, which beside causing low growth and protein production rate can negatively affect CQAs [8, 9]. On the other hand, concentrations higher than 40 mM stimulates cell division but also increases the accumulation of by-products (e.g. lactate, ammonia) that raise the stress level of the cells potentially altering the product quality [10, 11]. Therefore, glucose concentration has to be controlled to avoid too low and too high concentrations to couple relatively high productivity with good product quality.

Monoclonal antibody production with CHO cells is typically a fed-batch cultivation process when the consumed nutrients are added regularly to prolong cultivation time [4]. The fed-batch process begins with the batch phase when the initial concentrations of the media components decrease to a lower level as they are consumed. After a previously determined threshold is reached, the cultivation is fed (potentially multiple times) until its termination, that is the fed-batch phase of the process. The common practice nowadays for glucose concentration control is that glucose is administered either alone or together with other media components to maintain its concentration within a previously determined range. However, it is usually consumed in a high rate for the above-mentioned reasons, therefore, it is necessary to supply it at least daily but in small doses to avoid too high concentrations. Thus, glucose concentration fluctuates in relatively high frequency in a wide range. To determine the amount of glucose that is needed to be transferred to the bioreactor a sample has to be taken from the cultivation and analysed at-line afterwards. The frequent sampling poses a risk of contamination and general error compared to the fully automated controlling of other CPPs such as the pH or temperature. Furthermore, automated control enables tighter control of the CPP that is beneficial for process reproducibility. However, the on-line measurement of glucose, in contrast to these traditional CPPs requires a more advanced tool than for instance a relatively simple thermometer and these are not well established yet despite the PAT framework being released more than 10 years ago.

Near infrared (NIR) spectroscopy would be an ideal solution as it is non-destructive, fast and capable of on-line measurements with sterilisable, *in-situ* probes in bioreactors. Several studies were already published about the application of NIR spectroscopy to determine the concentration of glucose in aqueous fermentation and cell cultivation systems [12], even before the release of the PAT guidance [13, 14]. First, it was used for the monitoring of fermentation and cell cultivation processes at-line after withdrawing a sample from the reactor vessel and analysing the culture supernatant [13, 15–17] with benchtop analysers. The results were acceptably accurate to determine the glucose concentration and to monitor both type of processes, by reaching an error of prediction between 0.49 and 3.51 mM. A big step towards the original PAT goal was when Arnold *et al*. [14] used in-situ sterilisable probes to monitor cell cultivations in real-time, because the eventual goal of the automated control of the glucose concentration during the process could only be achieved if the NIR spectra acquisition is done real-time. Although, the conditions in cell cultivations are unfavourable for NIR spectroscopy, which was noted in the study of Arnold et al. [14], but their in-situ measurements could reproduce the accuracy of the previous at-line measurements. In fact, with an error of prediction of 0.53 mM the accuracy was close to the error of prediction of the reference method but 11 factors were used, which might indicate overfit. Henriques et al. achieved with five lab-scale reactors 1.84 mM error of prediction with only four factors [18]. However, Clavaud et al. [19] reached only 12.3 mM in industrial scale using the same number of factors as Henriques et al. In our view, the ideal error of prediction should not be higher than 5 mM. If the prediction error is not higher than this, it is acceptably accurate for the following reasons. Berry et al. [20] already published (although using Raman spectroscopy) about the establishment of an in-situ glucose controlling system. Their target concentration for control was 12.5 mM. If the prediction error is 5 mM deviation from the 12.5 mM in both directions could be tolerated by the cultivation; 7.5 mM is not low enough to cause starvation, while 17.5 mM is still low enough to prevent the formation of harmful by-products, as mentioned above. However, lower than 5 mM error would be desirable to achieve minimal deviation from the target concentration.

One of the biggest challenges in cell cultivation monitoring is water because the two water peaks, as a consequence of its strong NIR absorption, overshadow the peaks of analytes that are present in lower concentration, such as glucose. This is good if the goal is to determine the water content, which is often the case with agriculture-related measurements, but it is a disadvantage of NIR in bioprocess monitoring. In previous studies, attempts were made to minimize the effect of water by leaving the water-related regions of the spectra out of the analysis [21]. The selection of the appropriate regions (e.g. sensitive to glucose) could be done either by manual or by automated variable selection techniques such as genetic algorithms (GA) or interval Partial Least Squares (iPLS) for instance [18, 22, 23]. The saturated signal of the water peak (around 5000 cm^− 1^) was excluded from analysis and the other peak (around 7000 cm^− 1^) was excluded in another study [18, 19]. An additional challenge is that glucose concentration is low (typically lower than 40 mM, as mentioned earlier) throughout the cultivation, thus its NIR signal is weak and its intensity is continuously changing as the cells consume glucose. Automated variable selection could be favourable as opposed to manual because it could retrieve hidden correlations with specific parts of the spectra, which would be otherwise unnoticed due to the low concentration. In previous studies, various pre-processing techniques were applied to enhance the glucose signal to improve model performance [18, 19, 24]. First and second derivatives are the most commonly used because they successfully eliminate the baseline shift as well as increase the signal of analytes with lower concentration. The baseline shift is caused by the continuously changing cell density as the cultivation progresses [19]. Furthermore, cells are also responsible for NIR light scattering that is usually treated with standard normal variate (SNV) or multiplicative scatter correction (MSC) [18]. These pre-processing methods could also act as signal enhancers for the analyte by the reduction of the effect of light scattering in the spectra thus further improve model robustness. One of the most commonly used regression algorithms is PLS (Partial Least Squares) because it handles the multivariate data well and it weights each variable of the spectrum according to the variability detected in the spectra that corresponds with the variability in the reference data [25, 26]. Therefore, nowadays, PLS is the industry standard for NIR calibrations.

Proper variable selection and pre-processing could improve model performance but the wise selection of the calibration and validation dataset is also crucial. The spectral data is usually available from the monitoring of several bioreactor runs [18, 19]. The data might demonstrate run-to-run variability because of the process or the instrument and an additional source of variability is the difference between scales. To build a robust model the calibration samples have to be a good representation of the whole dataset thus they cover all the essential process variabilities. Typically, model robustness could be improved if more data is added to the calibration dataset because wider variability is covered with it, therefore, it is more likely that variabilities of the validation dataset are modelled [14]. To examine the covered variability of the dataset principal component analysis (PCA) is useful as it was demonstrated by Henriques et al. [18] The score plots are suitable tools to visualize the hidden variabilities in the data thus PCA can provide assistance to choose the calibration and validation dataset wisely [19]. However, most of the previous studies did not examine the scale-to-scale variabilities because they monitored only one scale [18, 19]. The effect of scale up and the usage of multiple scales in one global model was not evaluated previously.

In this study, the scale-up steps of a monoclonal antibody upstream process was monitored by NIR spectroscopy from shake flask scale to large production scale and the effect of adding more data of a cell cultivation technology under development was investigated. We aimed to reach similar or better accuracy of the glucose concentration prediction of the large production scale by NIR spectroscopy as previous studies by developing models using the data of each available scale and the combination of them.

The goal of this study was to investigate if NIR spectroscopy-based models could reach the accuracy of models based on other promising techniques (such as Raman spectroscopy) and create a benchmark for glucose concentration prediction during industrial scale CHO cell cultivations. This study also aimed to investigate the limitations of model scalability from shake flask scale to 5000 L scale.

## Materials and methods

### Shake flasks

#### Cell cultivation

An IgG1 monoclonal antibody producing Chinese hamster ovary (CHO) cell line (courtesy of Gedeon Richter, Plc.) was cultivated in fed-batch mode in four disposable, polycarbonate shake flasks (Corning Inc., Corning, NY, USA) with a working volume of 1000 ml. 280 ml commercially available, chemically defined, serum-free medium supplemented with L-Glutamine, L-Tyrosine and L-Phenylalanine (Sigma-Aldrich Co., St. Louis, MO, USA) to a concentration of 8, 1.2 and 2 mM, respectively, was seeded with 0.5–0.7 million cells/ml. The starting pH was set to 7.30 after inoculation and from day three it was maintained between 7.20 and 7.40 by adding 0.5 M sodium-carbonate solution daily during cultivation. Shake flasks were incubated in a Kühner incubator (Kühner AG, Basel, Switzerland) where parameters were set to a constant 37 °C and 5% CO_2_. Shake flasks were agitated with the built-in platform shaker at 110 rpm. Two shake flasks (SF1, SF2) were fed one time on cultivation day 3. Two shake flasks (SF3, SF4) were fed two times on cultivation day 3 and 5. The feeding medium volume was 15% of the actual cultivation medium volume at each feeding event. Both feeds were the same commercially available, chemically defined, serum and glucose-free composition. Additional 2 M glucose stock solution was added for SF3 and SF4 to adjust the glucose concentration to 8 mM on day 7. Shake flasks were sampled daily. Viable cell density and viability were determined with Countess™ Automated Cell Counter (Thermo Fisher Scientific, Waltham, MA, USA) and glucose concentration was measured with Nova StatStrip Xpress® Glucose meter (Nova Biomedical Corp., Waltham, MA, USA). The cultivations with one feed were run for 7 days and the cultivations with two feeds were run for 9 days until the concentration of glucose fell below 1.5 mM.

#### NIR spectra acquisition

Similarly to our previous study [27], after cultivation was terminated in the shake flasks, the glucose concentration was adjusted with a 2 M glucose stock solution to create a calibration ladder and NIR spectra were acquired, as well as samples for reference. The same instrument (see the specifications later) with the same settings and probe were used as with bioreactors, except the spectra acquisition was controlled with OPUS 6.5 (Bruker Optics GmbH, Ettlingen, Germany).

### Bioreactor scale-up

#### Cell cultivation

The same CHO cell line as used in the shake flask experiments was cultivated in fed-batch mode in 20 L (Bioengineering AG, Wald, Switzerland), 100 L (Bioengineering AG, Wald, Switzerland), 1000 L (Bioengineering AG, Wald, Switzerland) and 5000 L (Bioengineering AG, Wald, Switzerland) working volume, stainless-steel bioreactors. Commercially available chemically defined, serum-free cultivation medium was used, which was (similarly to shake flasks) supplemented with L-Glutamine, L-Tyrosine and L-Phenylalanine (Sigma-Aldrich Co., St. Louis, MO, USA) to a concentration of 8, 1.2 and 2 mM, respectively. Initial volume of 10 L, 60 L, 600 L, 3000 L of the media was seeded with 0.60 million cells/mL, respectively. All cultivations were maintained at 37.0 °C and the dissolved oxygen (DO) was controlled to 40%. The pH set-point was 7.15 and it was controlled with the addition of 10 m/m % phosphoric acid or 0.5 M sodium-carbonate solution. 20 L scale cultivations were fed two times, while 100 L, 1000 L and 5000 L scales were fed three times. All feeds used the same composition of commercially available, chemically defined, serum-free feed medium as used in the shake flask cultures. In addition to feeds, 2 M glucose stock solution was added daily after day 3 as glucose supplement as per the need of the cultures. Cultivations with two and three feeds were carried for 8 and 10 days, respectively. Cultivations were sampled daily. Viable cell density and viability were determined at-line with Countess™ Automated Cell Counter and glucose concentration was measured with Nova StatStrip Xpress® Glucose meter. Samples for off-line analysis were centrifuged in a bench-top centrifuge (3000 rpm, 10 min) and supernatants were kept frozen at − 20 °C until the measurements were performed.

#### NIR spectra acquisition

The NIR spectra were taken by a BRUKER Matrix-F (Bruker Optics GmbH, Ettlingen, Germany) FT-NIR system equipped with a TE InGaAs detector using an INGOLD IN271P immersion, transflection probe (2 mm optical pathlength), which was connected to the spectrometer via a 10 m long fibre optic cable. Each spectrum consisted an average of 256 scans, in the region of 11988.0–4297.0 cm^− 1^, with a resolution of 8 cm^− 1^. The equipment was the same during shake flask and bioreactor measurements but the method of spectra acquisition was different. During shake flask measurements, the probe was manually immersed into the cultivation media and cleaned with purified water between triplicates to avoid cross-contamination between samples and the instrument was controlled by OPUS 6.5 (Bruker Optics GmbH, Ettlingen, Germany). However, for bioreactor runs, one spectrum was acquired every 5 min during cultivation and the probe, which was coupled to the instrument with a fibre-optic cable dedicated to each reactor, was fitted in the culture vessels INGOLD port. The data collection was controlled by Simatic SiPAT v4.1 (Siemens AG, Munich, Germany). The spectra were very similar to previously published studies [18, 19], therefore, in this study they are not presented.

The cultivations, from where the spectral data is originating, were run with overlaps in a span of three years. The first experiments were 100 L scale then 20 L scale and 1000 L scale experiments were run in parallel with a 100 L scale experiment in the middle. The first 5000 L scale experiment (later used as test set) were run in parallel with the last two 1000 L scale experiments.

#### Glucose reference

The glucose concentration of the off-line samples was determined by CEDEX BioHT (Roche Diagnostics GmbH, Basel, Switzerland) measurements in triplicate after thawing the frozen sample supernatants at room temperature. This method has a standard deviation of 0.1 mM and 5% accuracy. Off-line data after averaging the triplicates served as reference data for model development and test.

### Data analysis

Spectra and reference data were imported to Matlab 9.1 (The MathWorks, Inc., Natick, MA, USA) but the actual multivariate data analysis was carried out with PLS_Toolbox 8.2.1 (Eigenvector Research, Inc., Manson, WA, USA).

First, Principal Component Analysis (PCA) was used to reduce the dimensions of the data to facilitate the discovery of hidden variabilities and uncover similarities and dissimilarities between spectra sets of experiments. Second, Partial Least Squares (PLS) regression models were developed to predict the glucose concentration based on NIR spectra. Prior to calibration, the spectra were aligned with reference data. Groups of three spectra (covering a 15 min range) nearest in time to the time of each sampling for reference were selected from the spectra of the whole bioreactor culture. According to calculations of our group, glucose consumption during those 15 min is negligible due to the slow metabolism of CHO cells. Therefore, the three spectra were assigned to the same reference data and they were used as parallel measurements. The first PLS model was created with the spectra of shake flask cultivations then spectra of four 20 L, three 100 L and seven 1000 L scale cultures were used for calibration that resulted four (single scale) PLS models. (Table 1) Afterwards, data from each run at the different scales were merged and two additional calibration models (combined scale) were developed as well. All PLS models were tested by predicting the glucose concentration of five, 5000 L scale runs. Model evaluation were based on the determination coefficient (*R*^2^) and Root-Mean Square Error of Prediction (RMSEP). Data were pre-processed with Savitzky–Golay derivative algorithm, Standard Normal Variate (SNV) or Multiplicative Scatter Correction (MSC) filters and mean centering (MC) scaling method and their combinations prior to calibration. Mean centering was always applied as last pre-processing step. Furthermore, variable selection was carried out using interval Partial Least Squares (iPLS) [28]. iPLS was used in forward mode, the step size was automatically selected, the number of latent variables were maximized in 15 and the two varied parameters were the number of intervals and the interval size between 1 and 25. The optimization goal of the pre-processing and variable selection was to achieve the highest *R*^2^ and lowest RMSEP of the PLS model based on the pre-treated spectra. The optimal number of latent variables, which were later used for the model, were selected in the range of 2–12 to obtain the lowest error of prediction. The option of cross-validation as a calibration evaluation tool was also examined but cross-validation was not representative to the test set, which is typical with bioprocess-related spectral data [29]. Therefore, instead of optimizing the cross-validation method, the test set of spectra was immediately used to evaluate the calibration model. The variable and pre-processing were adjusted if the results were not satisfactory but the test was maintained independent because the actual prediction affected the pre-treatments indirectly, after a prediction was run.

**Table 1:** Summary of the details of the PLS models

Cultivation scale	No. of spectra	No. of variables	Used spectral range	Pre-process^b^	No. of latent variables	R^2^C^c^	R^2^P^d^	RMSEC^e^ [mM]	RMSEP^f^ [mM]	RER^g^ [mM]
Shake flask	78	149	8169.2–7937.8; 7930.0–7837.5; 6433.5–6209.8; 6086.4–5399.8	MSC + mean center	7	0.975	0.512	1.41	14.95	1.91
20 L	217	400^a^	11987.6–11841.1; 11062.0–10761.1; 10599.1–10452.5; 9982.0–9681.1; 9056.3–8446.9; 8130.6–7984.0; 7667.8–7212.6; 6587.8–5669.8	MSC + mean center	4	0.742	0.668	3.80	5.31	5.27
100 L	206	149	8169.2–7937.8; 7930.0–7837.5; 6433.5–6209.8; 6086.4–5399.8	MSC + mean center	6	0.857	0.586	2.63	7.74	3.49
1000 L	297	149		MSC + mean center	9	0.920	0.676	2.58	5.79	5.00
20 L + 100 L	423	149		MSC + mean center	8	0.865	0.772	2.71	4.18	6.94
20 L + 100 L + 1000 L	720	149		MSC + mean center	11	0.895	0.803	2.64	4.32	6.94

^a^The 149 variables were also tested but 400 variables resulted in a more suitable model

^b^MSC is the abbreviation of the Multiplicative Scatter Correction pre-processing method

^c^*R*^2^ value of the calibration model

^d^*R*^2^ value of the prediction model

^e^Root-mean square error of calibration

^f^Root-mean square error of prediction

^g^Range error ratio

## Results and discussion

### PCA

**Fig. 1:**
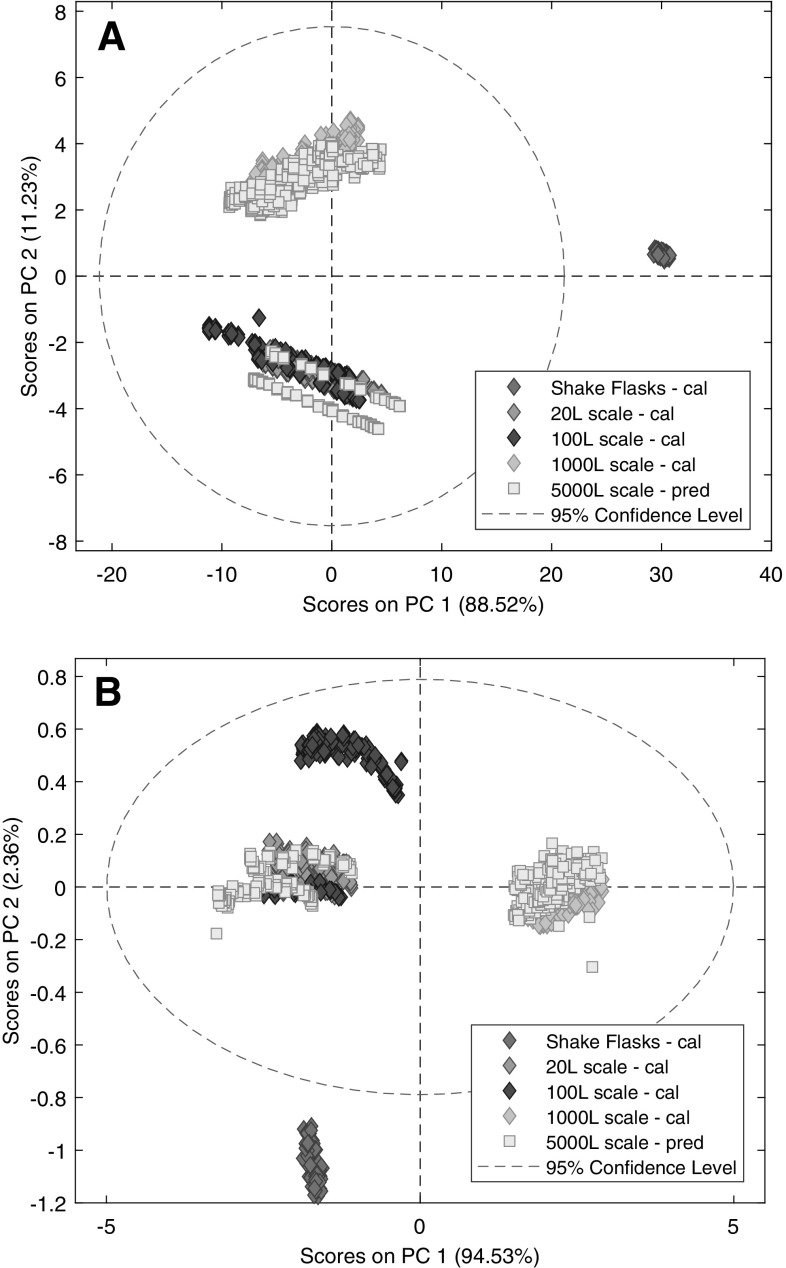
Score plots of the PCA models based on the full spectra after mean-center (**a**) or multiplicative scatter correction followed by mean-center (**b**) pre-processing

First step of data analysis was qualitative analysis using PCA as a pattern recognition tool. The first two PCA models were developed on the full spectra after mean-center and MSC followed by mean-center pre-processing. The first PC, irrespective of the pre-processing method, described the majority of the total variance in the data while the second PC described only a minor part (Fig. 1).

For mean-center pre-processing, the first PC corresponded to the variability between the shake flask and bioreactor cultivations (Fig. 1a). The scores of the shake flasks were gathered in one group with significant distance from the scores of bioreactor experiments in respect of PC1 and PC2 as well. Furthermore, shake flasks were in a compact group but bioreactor cultivations were more widely spread. Despite, that the elements of the cultivation technology (i.e. same media, fed-batch mode) and NIR instrument were similar for all experiments, the shake flask spectra were significantly different from bioreactor cultivations, according to the PCA model. Although the shake flask model system incorporates the light scattering effects of the cells, other perturbations were not modelled. These could be caused by, for example, the aeration (air bubbles) and the disintegration of cells (that cause more cell debris in the media) during the long cultivation process, while the whole cultivation is although gently but continuously agitated. The summarized effects of all non-modelled differences lead to the distance between shake flasks and bioreactors. Moreover, the compactness of the shake flasks group was the indication that shake flask experiments can be considered as snapshots of bioreactor cultivations, in respect of cell density and metabolite concentration. Therefore, spectra from shake flasks do not represent the progress of the cultivation as the spectra from bioreactors because they were acquired after the cultivation was terminated. Consequently, it was expected that the progress of the cultivation would not be represented by the shake flasks on the PCA score plot. The purpose of the shake flask experiments was not to model the cultivation progress but the cultivation environment especially the matrix. In theory, this should not be a problem regarding modelling because glucose concentration and cultivation progress is not coupled in fed-batch CHO cell cultivations.

The second PC described some minor scale-to-scale and run-to-run variability. Major technological differences or deviation from process parameter set points did not indicate the distance between bioreactor runs. Instead, the potential causes were presumable minor variations in the matrix composition and more likely the considerable alterations in the saturated water peak.

After MSC pre-processing was applied, the roles of PCs changed. The first PC still described the majority of variance, but it explained the scale-to-scale and run-to-run variability (Fig. 1b). In this case, the second PC corresponded to the difference between shake flasks and bioreactors. Furthermore, run-to-run variability between runs of the 100 L scale were also described by the second PC. Even the percent of explained variability was half of the percent of explained variability than the second PC of the previous PCA model, which means the outlying shake flasks contributed half as much to the model. This was the indication that MSC pre-processing was effectively decreased the variance between spectra of shake flasks and bioreactor runs. Therefore, there seemed to be a reasonable chance for acceptable quantitative model performance. However, the distance between shake flasks and the group of points of the 5000 L scale suggested that bias might occur in the model.

**Fig. 2:**
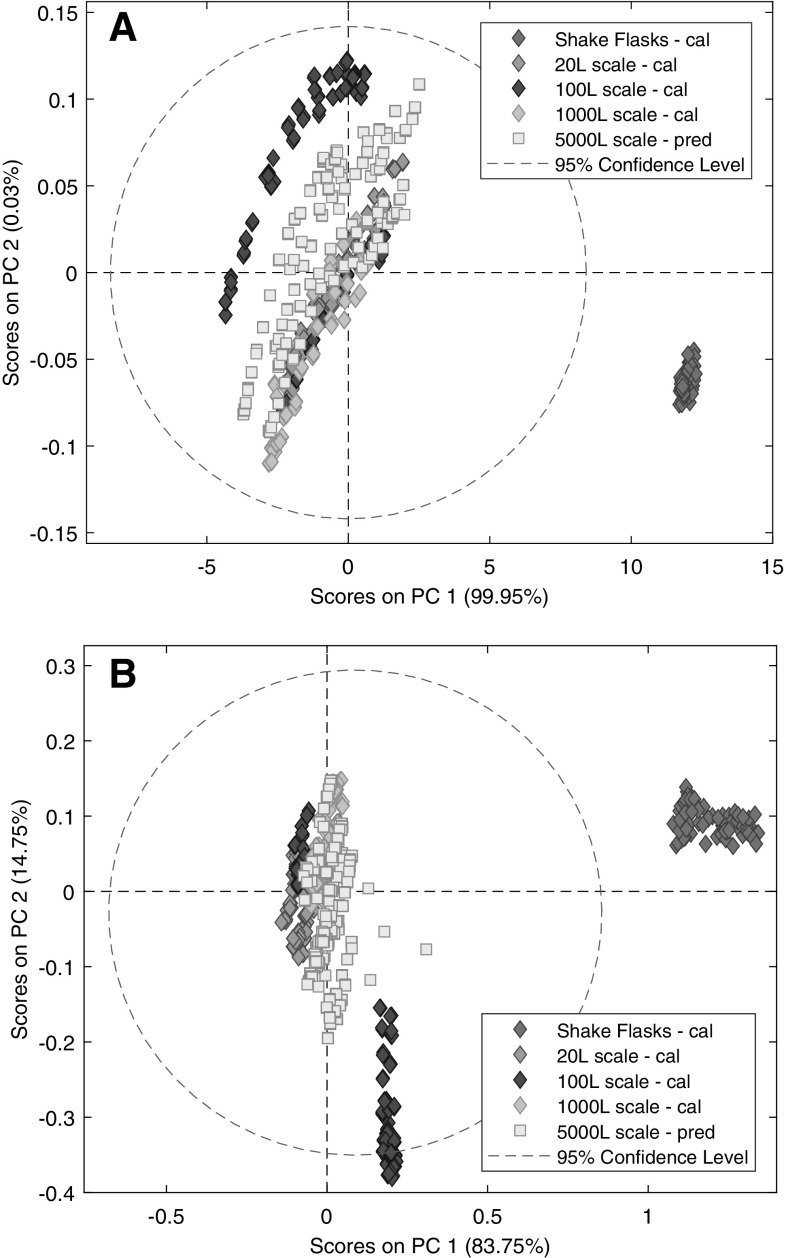
Score plots of the PCA models based on the 149 selected variables after mean-center (**a**) or multiplicative scatter correction followed by mean-center (**b**) pre-processing

The second two PCA models were built on the 149 selected variables (Table 1), subsequent to the PCA on the full spectra (Fig. 2). The 149 variables were selected by the iPLS algorithm in automatic method. For simple mean-center pre-processing, virtually all of the variance corresponded to the first PC again, which occurred between shake flasks and bioreactors. Instead of decreasing, elimination of the noisy variables of the spectra (e.g. water peaks), increased the contribution of the variability between shake flasks and bioreactors to the model. This was expressed by the higher percent of explained variance by the PC 1 compared to the PC 1 of the PCA model on the full spectra (Fig. 2a). However, variable selection resulted in the significant decrease of run-to-run variability and the contribution of the second PC to the model because the alterations in the saturated water peak (around 5000 cm^−1^) were eliminated. Therefore, acceptable quantitative modelling results were expected with bioreactors. After MSC followed by mean-center pre-processing, the percent of explained variance by the PC 1 were less than the PCA model on the full spectra (Fig. 2b). The first PC described the variance between shake flasks and bioreactors but also the deviation of a group of the points of the 100 L scale from the rest of the bioreactors. The deviance was also expressed by the second PC but it had neither technological nor significant spectroscopic indications, as mentioned earlier, therefore, it was not excluded from the dataset for further analysis. Nevertheless, the bioreactor runs located more closely together after pre-processing, which meant that the conditions were appropriate for prediction of the 5000 L scale.

### PLS

#### Shake flasks

**Fig. 3:**
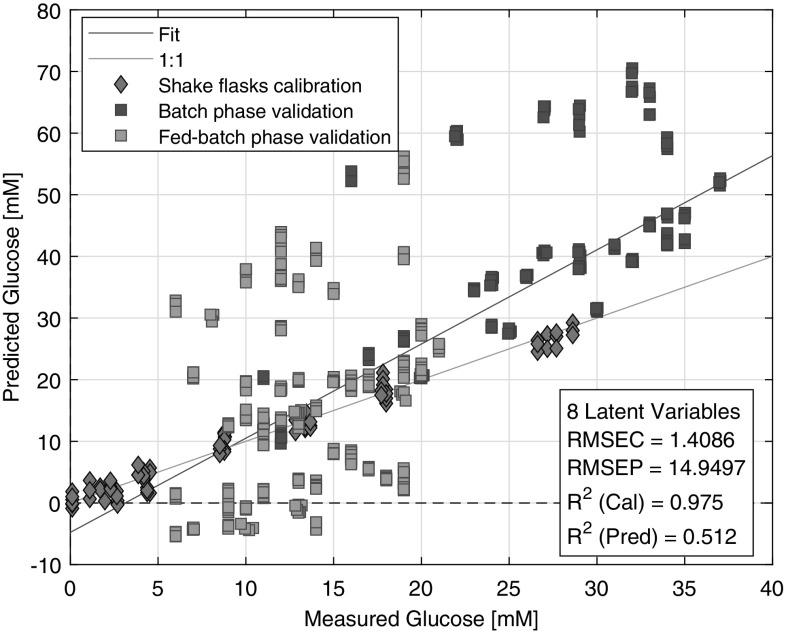
Measured vs. Predicted plot of the shake flask-based glucose concentration model

The first PLS model was built using the spectra of shake flasks to predict the glucose concentration of the five 5000 L scale runs. Shake flask-based models could determine the glucose concentration, which was indicated by an error of calibration lower than 1.5 mM and *R*^2^ higher than 0.97 (Fig. 3). However, prediction performance was arguably satisfied the criteria of a good prediction because predicted values scattered inaccurately in the batch and fed-batch phase as well (Fig. 3). The calibration range covered the glucose concentration range of the bioreactor experiments but the general difference, which was observed with the PCA, between spectra of shake flasks and bioreactors caused that the shake flask based model is non-transferable to larger scales. (Table 1) Therefore, the shake flask model system seemed to be good to test the limits of in-situ, on-line NIR spectroscopy in CHO cultivations but it does not model adequately an actual bioreactor cultivation. The reasons of the model inaccuracy were detailed in our previous study [30]. However, the model building workflow was different in this study from the workflow of the previous study because the model (i.e. pre-processing, variable selection, latent variable numbers) was optimized to the prediction set. Thus, although the expectations were not high, the results of this model were inserted for comparison purposes.

#### Single scale models

**Fig. 4:**
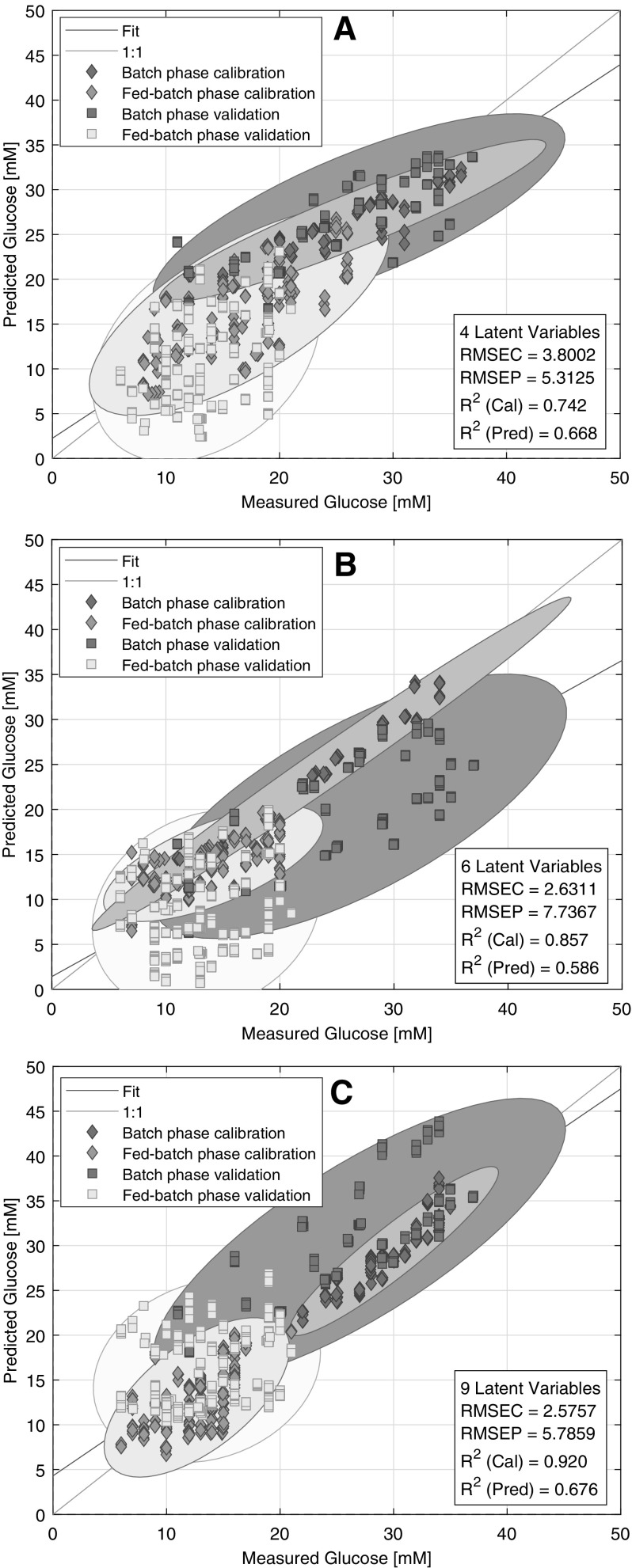
Measured vs. Predicted plots of the 20 L bioreactor (**a**), 100 L bioreactor (**b**), 1000 L bioreactor (**c**) based glucose concentration models. The ellipses around points are the 95% confidence ellipses of the points in the given class. The ellipses are used to visualize the overlapping of different cultivation phases

After the shake flask-based model, the spectra that were acquired in different bioreactor scales were also tested for modelling. Prediction performance (*R*^2^, RMSEP) improved significantly if the model was based on the 20 L scale bioreactor data. (Table 1) However, a dichotomy between the batch and fed-batch phases were displayed. The model was more accurate during the batch phase and predicted points below 20 mM were scattered during the fed-batch phase. To put the 5.3 mM RMSEP into context, the model could not differentiate between 18 and 5 mM, based on the measured vs. predicted plot (Fig. 4a). This amount of data might have been not sufficient to model the predicted data and the inaccuracy could be explained by the very weak NIR signal of glucose due to the low concentrations below 20 mM.

The model based on the 100 L scale data exhibited similar characteristics to the model of the 20 L scale in terms of prediction accuracy. However, the slope of the regression line and the test points diverted significantly from the target line (Fig. 4b). One 100 L scale run was a minor spectroscopic outlier, as could be observed with PCA (Fig. 2), that could be a possible explanation of the model performance because the outlying run could cause a diversion in the model that emerged in prediction.

The third scale that was used for modelling was the 1000 L scale. The calibration results were similar to the 100 L scale but the prediction results were closer to the more accurate 20 L scale (Fig. 4C). However, this scale also showed an offset for one of the 5000 L runs that the PCA did not forecast. It is more likely that the model could not explain the variabilities between the 5000 L runs, which was emphasized by the prediction.

#### Combined scale models

**Fig. 5:**
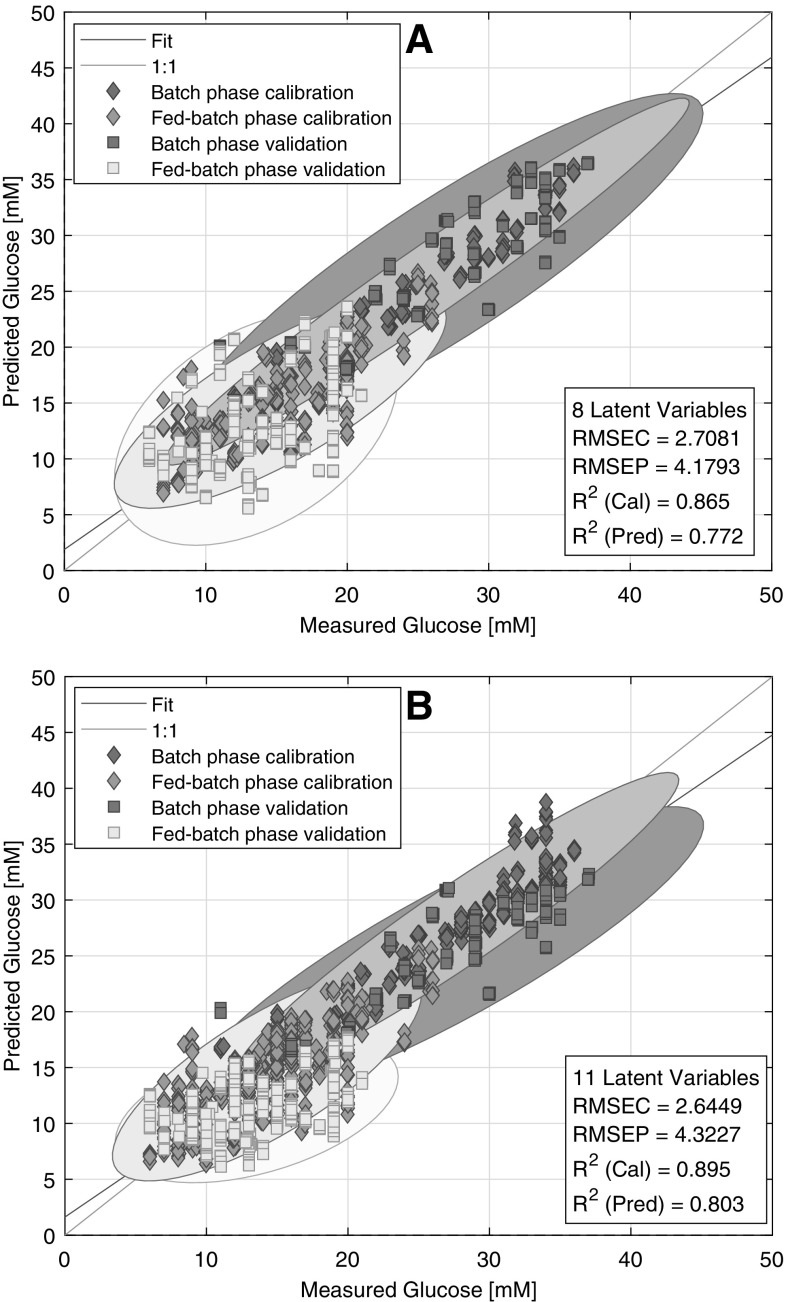
Measured vs. Predicted plots of the 20 L and 100 L scale (**a**), the 20 L, 100 L and 1000 L scale (**b**) bioreactors based glucose concentration models. The ellipses around points are the 95% confidence ellipses of the points in the given class. The ellipses are used to visualize the overlapping of different cultivation phases

To broaden the covered variability of the models, and thus achieve predictions that are more accurate, the data of the single scale models were combined and used for modelling subsequently. It was expected that more data from various scales would result in predictions that are more robust, with lower RMSEP and without offsets. However, the spectra of shake flasks were not incorporated with the bioreactor spectra; because of the poor single results it was not expected that calibrations could be improved. The data of the 20 L, 100 L and 1000 L scale were merged in every combination and a PLS model was fitted subsequently. The combination of the 20 L and 100L scale data resulted the lowest error of prediction amongst other models, supposedly because this dataset covers the variability of the test data most accurately. (Table 1) The 4.18 mM RMSEP indicated an acceptably accurate prediction and the points were scattered along the regression line in a narrow range. However, the model could not differentiate between 10 mM and 20 mM during the fed-batch phase that could be observed on the measured vs. predicted plot (Fig. 5a). This could be the limit of accuracy for the NIR technique in this environment. The model was also tested without the deviating groups of spectra (Fig. 2b) of the 100 L scale but the results did not improve (data not shown).The combination of the 20 L and 1000 L and the 100 L and 1000 L scale runs displayed even higher scattering at lower concentrations, therefore, the results are not shown.

To try to cover an even wider variability and to achieve a more robust prediction, the data of the 1000 L scale was merged with the data of the 20 L and 100 L scales. The performance of the two models could be considered the same with similar accuracy. (Table 1) No significant offset could be observed either during the batch phase or during the fed-batch phase (Fig. 5b). However, in contrast to the previously discussed model, now the optimal number of latent variables was eleven instead of eight. This indicated that this PLS model had to describe even more variability than the previous model, and the more data did not produce a more accurate model. Instead, the prediction error was slightly higher and it is advisable to always use a simpler model, if possible.

#### Glucose concentration during cultivation

**Fig. 6:**
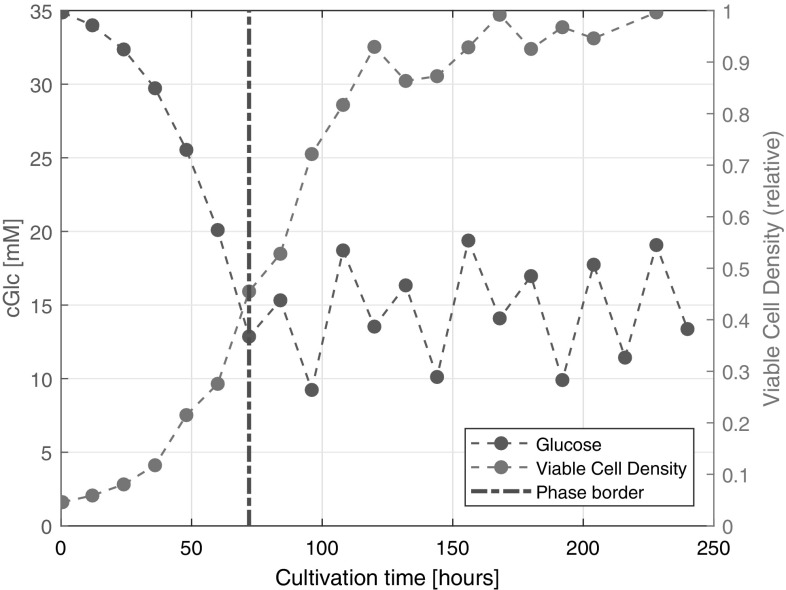
The offline measured glucose concentration (left axis) and atline viable cell density and viability (right axis), relative to the highest viable cell density and viability during one of the 5000 L cultivations. The phase border line indicates the shift between the batch and the fed-batch phase of the cultivation. Only data of one run is plotted as an illustration. Every other runs have the same characteristic

If the scattering of the predicted points at every calibration model is analysed, it could be observed that the predicted points scattered in a wider range at lower and narrower range at higher concentrations of glucose. This pattern is due to the phase of the cultivation because every fed-batch cultivation can be divided to two phases according to feed additions (Fig. 6). NIR spectroscopy is challenged differently in each cultivation phase that could be a reasonable explanation for the behaviour of the above-discussed prediction models. A cultivation begins with the batch phase after inoculation when, after a short accommodation period, cells start to grow rapidly while the concentration of the initial high media components decreases. The reduction of the glucose concentration is an indication of the cell proliferation. The batch phase is the chemically most homogenous part of the cultivation process because prior to the first feed addition, only the components of the media are present and the concentration of metabolites and products is low because nutrients are mostly turned into biomass. Moreover, concentration of the media components is the highest during the batch phase resulting in the strongest spectroscopic signals. Conversely, cell density is lower during the batch phase, thus optical density and light scattering effect of the cells is also low. Furthermore, the light scattering effect of cell debris is small because cell viability is high. Therefore, glucose signals are not suppressed by the optical characteristics of the cells. Instead, the increase in optical density caused by the increasing cell density supports the determination of glucose because it cross-correlates with the decrease of glucose concentration

## Conclusions

In this study, results of glucose concentration monitoring by NIR spectroscopy during a complete scale up of a mammalian cell cultivation process were presented and discussed. The PCA score plots revealed that after pre-processing the bioreactor cultivations could be regarded as very similar but shake flask cultivations differ significantly from them based on the NIR spectra. This was also verified with the PLS models, where the shake flask based model predicted the test dataset with a 15 mM error of prediction but error of prediction for bioreactor data based models were less than one-third of the error of prediction for the shake flask based. Different scales were all used for monitoring glucose concentration but the lowest RMSEP (4.18 mM) was achieved when the 20 L and 100 L scale dataset were merged and used together for model building. This dataset incorporated all the necessary variabilities for an accurate model but it did not contained too much noise (subtle changes and natural variability of cell culture) that would make the prediction inaccurate. However, the magnitude of the error of prediction depended on the cultivation phase. NIR based glucose prediction performed more accurately in the batch phase, when the decrease in the glucose concentration is highly correlated with the increase in cell density. In the fed-batch cultivation phase, when glucose concentration is lower and uncorrelated with other factors the models became inaccurate. Therefore, it was concluded that the error of prediction for NIR based models could be the same as reported in previous studies with other techniques. However, the low RMSEP value of the NIR based prediction was an average value for the entire cultivation and the NIR sustained several limitations during different cultivation phases. This is the main drawback of the NIR technique because accuracy of glucose concentration monitoring is more important during the fed batch phase when feeds are added to the culture. Therefore, NIR for glucose monitoring could only be applied with restrictions in contrast to other more suitable monitoring techniques.

## References

[1] Wurm FM (2004). Production of recombinant protein therapeutics in cultivated mammalian cells. Nat Biotech.

[2] Li J, Zhu Z (2010). Research and development of next generation of antibody-based therapeutics. Acta Pharmacol Sin.

[3] Dumont J, Euwart D, Mei B, Estes S, Kshirsagar R (2016). Human cell lines for biopharmaceutical manufacturing: history, status, and future perspectives. Crit Rev Biotechnol.

[4] Xie L, Zhou W, Ozturk S, Hu W-S (2005). Fed-batch cultivation of mammalian cells for the production of recombinant proteins. Cell culture technology for pharmaceutical and cell-based therapies.

[5] U.S. Food and Drug Administration (2004) PAT—a framework for innovative pharmaceutical development, manufacturing, and quality assurance. https://www.fda.gov/downloads/Drugs/%E2%80%A6/Guidances/ucm070305.pdf. Accessed 25 Feb 2019

[6] Joan Cairó J, Gódia F, Ozturk S, Hu W-S (2005). Cell metabolism. Cell culture technology for pharmaceutical and cell-based therapies.

[7] Liu B, Spearman M, Doering J, Lattova E, Perreault H, Butler M (2014). The availability of glucose to CHO cells affects the intracellular lipid-linked oligosaccharide distribution, site occupancy and the N-glycosylation profile of a monoclonal antibody. J Biotechnol.

[8] Liu B, Spearman M, Doering J, Lattová E, Perreault H, Butler M (2014). The availability of glucose to CHO cells affects the intracellular lipid-linked oligosaccharide distribution, site occupancy and the *N*-glycosylation profile of a monoclonal antibody. J Biotechnol.

[9] Lu S, Sun X, Zhang Y (2005). Insight into metabolism of CHO cells at low glucose concentration on the basis of the determination of intracellular metabolites. Process Biochem.

[10] Liu Z, Dai S, Bones J, Ray S, Cha S, Karger BL, Li JJ, Wilson L, Hinckle G, Rossomando A (2015). A quantitative proteomic analysis of cellular responses to high glucose media in Chinese hamster ovary cells. Biotechnol Progr.

[11] Aghamohseni H, Ohadi K, Spearman M, Krahn N, Moo-Young M, Scharer JM, Butler M, Budman HM (2014). Effects of nutrient levels and average culture pH on the glycosylation pattern of camelid-humanized monoclonal antibody. J Biotechnol.

[12] Abu-Absi NR, Martel RP, Lanza AM, Clements SJ, Borys MC, Li ZJ (2014). Application of spectroscopic methods for monitoring of bioprocesses and the implications for the manufacture of biologics. Pharm Bioprocess.

[13] Rhiel M, Cohen MB, Murhammer DW, Arnold MA (2002). Nondestructive near-infrared spectroscopic measurement of multiple analytes in undiluted samples of serum-based cell culture media. Biotechnol Bioeng.

[14] Arnold SA, Crowley J, Woods N, Harvey LM, McNeil B (2003). In-situ near infrared spectroscopy to monitor key analytes in mammalian cell cultivation. Biotechnol Bioeng.

[15] Riley M-R, Rhiel M, Zhou X, Arnold M-A, Murhammer D-W (1997). Simultaneous measurement of glucose and glutamine in insect cell culture media by near infrared spectroscopy. Biotechnol Bioeng.

[16] Riley MR, Crider HM, Nite ME, Garcia RA, Woo J, Wegge RM (2001). Simultaneous measurement of 19 components in serum-containing animal cell culture media by Fourier transform near-infrared spectroscopy. Biotechnol Progr.

[17] Riley MR, Okeson CD, Frazier BL (1999). Rapid calibration of near-infrared spectroscopic measurements of mammalian cell cultivations. Biotechnol Progr.

[18] Henriques JG, Buziol S, Stocker E, Voogd A, Menezes JC (2009). Monitoring mammalian cell cultivations for monoclonal antibody production using near-infrared spectroscopy. Adv Biochem Eng Biotechnol.

[19] Clavaud M, Roggo Y, Von Daeniken R, Liebler A, Schwabe JO (2013). Chemometrics and in-line near infrared spectroscopic monitoring of a biopharmaceutical Chinese hamster ovary cell culture: prediction of multiple cultivation variables. Talanta.

[20] Berry BN, Dobrowsky TM, Timson RC, Kshirsagar R, Ryll T, Wiltberger K (2016). Quick generation of Raman spectroscopy based in-process glucose control to influence biopharmaceutical protein product quality during mammalian cell culture. Biotechnol Progr.

[21] Hakemeyer C, Strauss U, Werz S, Jose GE, Folque F, Menezes JC (2012). At-line NIR spectroscopy as effective PAT monitoring technique in Mab cultivations during process development and manufacturing. Talanta.

[22] Mehmood T, Liland KH, Snipen L, Sæbø S (2012). A review of variable selection methods in partial least squares regression. Chemometr Intell Lab.

[23] Xiaobo Z, Jiewen Z, Povey MJ, Holmes M, Hanpin M (2010). Variables selection methods in near-infrared spectroscopy. Anal Chim Acta.

[24] Lopes JA, Costa PF, Alves TP, Menezes JC (2004). Chemometrics in bioprocess engineering: process analytical technology (PAT) applications. Chemometr Intell Lab.

[25] Rathore A, Singh S (2015). Use of multivariate data analysis in bioprocessing. BioPharm Int.

[26] Wold S, Sjöström M, Eriksson L (2001). PLS-regression: a basic tool of chemometrics. Chemometr Intell Lab.

[27] Kozma B, Párta L, Zalai D, Gergely S, Salgó A (2014). A model system and chemometrics to develop near infrared spectroscopic monitoring for Chinese hamster ovary cell cultivations. J Near Infrared Spectros.

[28] Interval PLS (IPLS) for Variable Selection (2011) http://wiki.eigenvector.com/index.php?title=Interval_PLS_(IPLS)_for_Variable_Selection. Accessed Nov 2017

[29] Berry B, Moretto J, Matthews T, Smelko J, Wiltberger K (2015). Cross-scale predictive modeling of CHO cell culture growth and metabolites using Raman spectroscopy and multivariate analysis. Biotechnol Prog.

[30] Kozma B, Hirsch E, Gergely S, Párta L, Pataki H, Salgó A (2017). On-line prediction of the glucose concentration of CHO cell cultivations by NIR and Raman spectroscopy: comparative scalability test with a shake flask model system. J Pharmaceut Biomed Anal.

